# Robust Multimodal Deep Learning for Lymphoma Subtype Classification Using ^18^F-FDG PET Maximum Intensity Projection Images and Clinical Data: A Multi-Center Study

**DOI:** 10.3390/cancers18020210

**Published:** 2026-01-09

**Authors:** Seonhwa Kim, Jun Hyeong Park, Chul-Ho Kim, Seulgi You, Jeong-Seok Choi, Jae Won Chang, In Young Jo, Byung-Joo Lee, Il-Seok Park, Han Su Kim, Yong-Jin Park, Jaesung Heo

**Affiliations:** 1 Department of Radiation Oncology, School of Medicine, Ajou University, Suwon 16499, Republic of Korea; 2 Department of Biomedical Sciences, Graduate School, Ajou University, Suwon 16499, Republic of Korea; 3 Department of Otolaryngology, School of Medicine, Ajou University, Suwon 16499, Republic of Korea; 4 Department of Radiology, School of Medicine, Ajou University, Suwon 16499, Republic of Korea; 5 Department of Otorhinolaryngology—Head and Neck Surgery, College of Medicine, Inha University, Incheon 22212, Republic of Korea; 6 Department of Otolaryngology—Head and Neck Surgery, Chungnam National University Hospital, Daejeon 35015, Republic of Korea; 7 Department of Radiation Oncology, Soonchunhyang University Hospital, Cheonan 33019, Republic of Korea; 8 Department of Otorhinolaryngology—Head and Neck Surgery, College of Medicine, Pusan National University and Biomedical Research Institute, Pusan National University Hospital, Pusan National University, Busan 49241, Republic of Korea; 9 Department of Otorhinolaryngology—Head and Neck Surgery, Hallym University Dongtan Sacred Heart Hospital, Hallym University College of Medicine, Chuncheon 24252, Republic of Korea; 10 Department of Otorhinolaryngology—Head and Neck Surgery, School of Medicine, Ewha Womans University, Seoul 03760, Republic of Korea; 11 Department of Nuclear Medicine, Ajou University Medical Center, School of Medicine, Ajou University, Suwon 16499, Republic of Korea

**Keywords:** cancer imaging, deep learning, Hodgkin lymphoma, b-cell lymphoma, ^18^F-FDG, PET image

## Abstract

**Background:** Previous attempts to classify lymphoma subtypes based on metabolic features extracted from ^18^F-FDG PET imaging have been hindered by inconsistencies in imaging protocols, scanner types, and inter-institutional variability. To overcome these limitations, we propose a multimodal deep learning framework that integrates harmonized PET imaging features with structured clinical information. The proposed framework is designed to perform hierarchical classification of clinically meaningful lymphoma subtypes through two sequential binary classification tasks. **Methods:** We collected multi-center data comprising ^18^F-FDG PET images and structured clinical variables of patients with lymphoma. To mitigate domain shifts caused by different scanner manufacturers, we integrated a Scanner-Conditioned Normalization (SCN) module, which adaptively harmonizes feature distributions using manufacturer-specific parameters. Performance was validated using internal and external cohorts, with the statistical significance of performance gains assessed via DeLong’s test and bootstrap-based CI analysis. **Results:** The proposed model achieved an area under the curve (AUC) of 0.89 (internal) and 0.84 (external) for Hodgkin lymphoma versus non-Hodgkin lymphoma classification and 0.84 (internal) and 0.76 (external) for diffuse large B-cell lymphoma versus follicular lymphoma classification (*p* > 0.05). These results were obtained using a multimodal model that integrated anterior and lateral maximum intensity projection (MIP) images with clinical data. **Conclusions:** This study demonstrates the potential of a deep learning-based approach for lymphoma subtype classification using non-invasive ^18^F-FDG PET imaging combined with clinical data. While further validation in larger, more diverse cohorts is necessary to address the challenges of rare subtypes and biological heterogeneity, LymphoMAP serves as a meaningful step toward developing assistive tools for early clinical decision-making. These findings underscore the feasibility of using automated pipelines to support, rather than replace, conventional diagnostic workflows in personalized lymphoma management.

## Simple Summary

Lymphoma subtypes require different therapeutic strategies. However, accurate classification is challenging due to variable imaging phenotypes and patient characteristics. Although histopathology is the gold standard, noninvasive fluorine-18 fluorodeoxyglucose (^18^F-FDG) positron emission tomography (PET) provides complementary subtype-specific information. In this study, we developed a deep learning model integrating ^18^F-FDG PET images with structured clinical data. We applied harmonization techniques to multi-institutional datasets from six centers. The model achieved 89% (internal) and 84% (external) accuracy in distinguishing Hodgkin from non-Hodgkin lymphoma. This approach improves diagnostic consistency and reproducibility, facilitates early subtype assessment prior to histopathological confirmation, and holds promise for broader applications in imaging-based disease classification.

## 1. Introduction

Lymphoma is a heterogeneous malignant disease comprising more than 80 subtypes, with considerable histological variability even within individual subtypes [1,2]. Therefore, diagnosis based solely on morphological observation is limited, necessitating the use of complementary molecular diagnostic techniques, such as tissue biopsy, immunohistochemical staining, and genetic rearrangement testing [3]. The diagnostic process is expensive, time-consuming, and repetitive. This delays clinical decision-making. To overcome these difficulties, automated subtype classification systems using fluorine-18 fluorodeoxyglucose (^18^F-FDG) positron emission tomography (PET) images have been actively studied [4].

^18^F-FDG PET is widely used as a standard imaging modality for lymphoma and plays a crucial role in disease staging and treatment response assessment. Because FDG uptake patterns reflect underlying tumor biology, metabolic heterogeneity captured on PET provides clinically meaningful cues that differ across lymphoma subtypes [5]. Accordingly, multiple studies have attempted to classify lymphoma subtypes by exploiting these metabolic signatures on PET images [6,7]. In particular, a nuclear medicine imaging-based approach was used to extract quantitative features from PET images, and existing machine learning algorithms were applied to subtype prediction. These methods rely on complex, operator-dependent preprocessing and manual feature engineering. This limits their reproducibility and scalability across clinical settings.

Recent advances in deep learning have led to growing interest in automatically learning the metabolic characteristics embedded in PET images and leveraging these features for lesion detection and treatment response prediction in patients with lymphoma [8,9]. However, most existing deep learning studies rely on single-center datasets and inadequately address heterogeneity arising from differences in scanner hardware, imaging protocols, and reconstruction algorithms [10]. In multi-center settings, such variations can introduce systematic biases that significantly impair the generalizability and robustness of predictive models [11,12].

Based on this background, we integrated the statistical harmonization principles of ComBat directly into the deep learning framework by introducing a Scanner-Conditioned Normalization (SCN) module to mitigate batch effects arising from multi-center PET data during model training. This design allows scanner manufacturer-related variability to be explicitly regulated within the training process, thereby facilitating robust lymphoma subtype classification across diverse equipment manufacturers. The proposed model, LymphoMAP (lymphoma multi-modality AI for PET), is designed to automatically classify lymphoma subtypes by leveraging both ^18^F-FDG PET maximum intensity projection (MIP) images and clinical data. In this study, using the same model architecture, two binary classification tasks were performed sequentially: (1) Hodgkin lymphoma (HL) and non-Hodgkin lymphoma (NHL) classification and (2) representative NHL subtype classification (diffuse large B-cell lymphoma [DLBCL] and follicular lymphoma [FL]).

## 2. Materials and Methods

### 2.1. Study Design and Patient Selection

**Figure 1:**
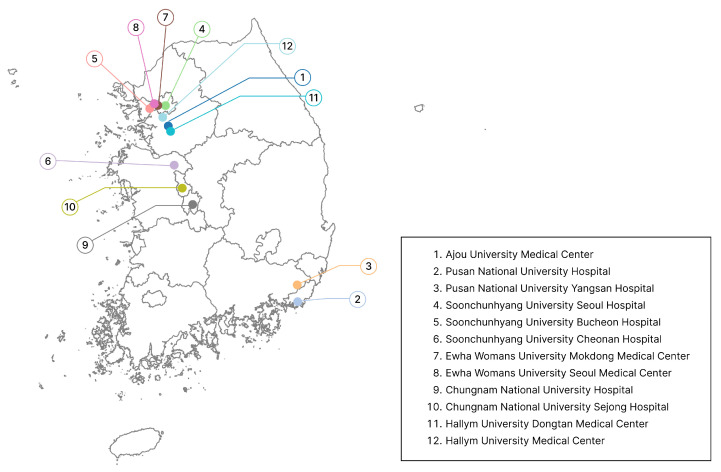
Geographic distribution of participating hospitals.

We conducted a retrospective analysis of patients diagnosed with lymphoma between 1 January 2000 and 31 December 2022 across 12 hospitals affiliated with six universities in the Republic of Korea, including both main and branch hospitals. Participating institutions included Ajou University Medical Center, Pusan National University Hospital (PNUH), Soonchunhyang University Medical Center, Chungnam National University Hospital, Ewha Womans University Medical Center, and Hallym University Medical Center. The geographical distribution of the participating hospitals is shown in Figure 1.

**Figure 2:**
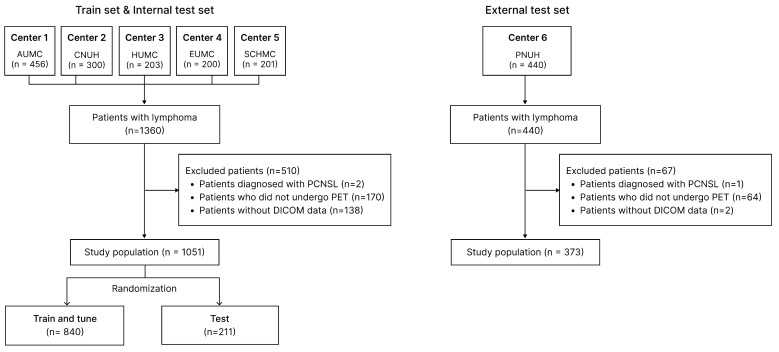
Flow diagram of a multi-center cohort study. Abbreviation: AUMC, Ajou University Medical Center; CNUH, Chungnam National University Hospital; EUMC, Ewha Womans University Medical Center; HUMC, Hallym University Medical Center; PNUH, Pusan National University Hospital; SCHMC, Soonchunhyang University Hospital; and PCNSL, primary central nervous system lymphoma.

Among the six institutions, an external test institution (PNUH) was selected based on data completeness (Supplementary Table S1) [13]. Data from five institutions were randomly categorized at the patient level into development (80%) and holdout internal test datasets (20%). The model was trained and tuned using the training dataset, and its final performance was evaluated using the internal and external test sets. Histopathological diagnosis of lymphoma was based on biopsy or lymph node dissection. Patients diagnosed with primary central nervous system lymphoma were excluded due to distinct clinical and pathological characteristics and treatment approaches that differed from those of the systemic lymphoma subtypes targeted in this study. Patients without pretreatment PET images or Digital Imaging and Communication in Medicine (DICOM) metadata were excluded. The patient selection flowchart for this study is shown in Figure 2. This study was approved by the Institutional Review Board of Ajou University Hospital (AJOUIRB-MDB-2022-248), which waived the requirement for informed consent because it was a retrospective study.

### 2.2. Data Preprocessing

For each patient, ^18^F-FDG PET scans performed for pretreatment staging were included in the analysis, with a single PET scan per patient used for model training and evaluation. Imaging data were acquired across 12 hospitals (six university hospitals and their affiliated branches), using 15 PET/computed tomography (CT) scanner models from four different manufacturers. PET/CT images were acquired using scanners from major vendors, including GE Healthcare (Chicago, IL, USA), Siemens Healthineers (Erlangen, Germany), Philips Medical Systems (Amsterdam, The Netherlands), and CTI PET Systems (Knoxville, TN, USA; now part of Siemens Healthineers). All scans were performed approximately 60 min after the intravenous administration of ^18^F-FDG, following each institution’s clinical imaging protocol. Reconstruction algorithms, slice thickness, and other acquisition parameters varied depending on the scanner and institution. Detailed specifications are provided in Supplementary Table S2.

Raw pixel values were converted to physical units using the RescaleSlope and RescaleIntercept parameters from the DICOM metadata. Attenuation correction was applied based on the scan start time. Standardized uptake value was calculated using the patient’s weight and the administered dose of ^18^F-FDG. MIP images were generated from both anterior and lateral views. Each image was resized such that its longest side was scaled to 310 pixels, followed by zero padding to obtain a uniform image size of 310 × 310 pixels. All images were converted to grayscale and replicated across three channels. Pixel values were independently normalized to a range of 0–1 for each image using its own minimum and maximum pixel values.

Clinical variables included demographic information, laboratory test results, and clinical risk factors. Specifically, the dataset comprised age, Ann Arbor stage [14], Deauville score, white blood cell count (WBC), absolute neutrophil count (ANC), absolute lymphocyte count (ALC), platelet count (PLT), hemoglobin level (Hb), neutrophil-to-lymphocyte ratio (NLR), platelet-to-lymphocyte ratio (PLR), and lactate dehydrogenase (LDH) level. The Deauville score, a 5-point scale based on ^18^F-FDG uptake intensity in the mediastinum and liver, was used to evaluate the metabolic activity on PET/CT scans. Additionally, binary indicators were included for family history of cancer, hypertension, abnormal blood glucose levels, and smoking history. Categorical variables were binarized, and continuous variables were normalized using statistics derived from the training dataset, which were consistently applied to both internal and external test datasets.

### 2.3. Model Architecture for Subtype Classification

In this study, we propose LymphoMAP, a multimodal deep learning model that integrates images and clinical variables for lymphoma subtype classification. LymphoMAP comprises an image feature extractor, a clinical feature extractor, and a classifier. For image feature extraction, a convolutional neural network (CNN)-based backbone pretrained on ImageNet was used [15]. The image feature extractor architecture was selected through comparative experiments among pretrained models—ResNet-50, EfficientNetV2-S, and ConvNeXt-Small—which were chosen to represent distinct and widely used CNN architectural paradigms with different performance–efficiency trade-offs. The model with the best performance was selected as the final backbone. To ensure a fair and transparent comparison across all comparative experiments conducted in this study, all models were trained and evaluated under identical data partitions, architectures, and training protocols, following established principles of fair benchmarking in prior comparative studies [16].

The input image was converted into a high-dimensional embedding vector through a CNN backbone. To minimize the batch effects among ^18^F-FDG PET MIP images acquired using scanners from different manufacturers, an SCN module inspired by the ComBat harmonization principle was implemented as a trainable module within the deep learning architecture. Unlike the traditional post-hoc statistical ComBat, this module was integrated directly into the deep learning architecture as a trainable module. Specifically, the module first standardizes feature embeddings to suppress manufacturer-specific statistical biases and then applies learnable scale (γ) and shift (β) parameters retrieved from manufacturer-conditioned embeddings. By conditioning the normalization process on scanner manufacturer information, the model dynamically harmonizes feature distributions in an end-to-end manner, thereby enhancing robustness and generalizability for lymphoma subtype classification. Equation 1 illustrates the proposed SCN mechanism. First, the input feature x is standardized using instance-wise statistics ($\mu \left(x\right)$ and σ(x)) via Layer Normalization. This removes sample-specific intensity variations. Next, the feature distribution is recalibrated using manufacturer-specific parameters (γ_s_ and β_s_). These are conditioned on the manufacturer index s and learned jointly with CNN parameters via backpropagation. The overall process is summarized as Equation (1), where ϵ is set to 1 × 10^−6^ for numerical stability.

(1) $$\hat{x}=\frac{x-\mu \left(x\right)}{\sqrt{\sigma {\left(x\right)}^{2}+\epsilon }},\;\;y=\hat{x}\cdot \gamma_{s}+\beta_{s}$$

**Figure 3:**
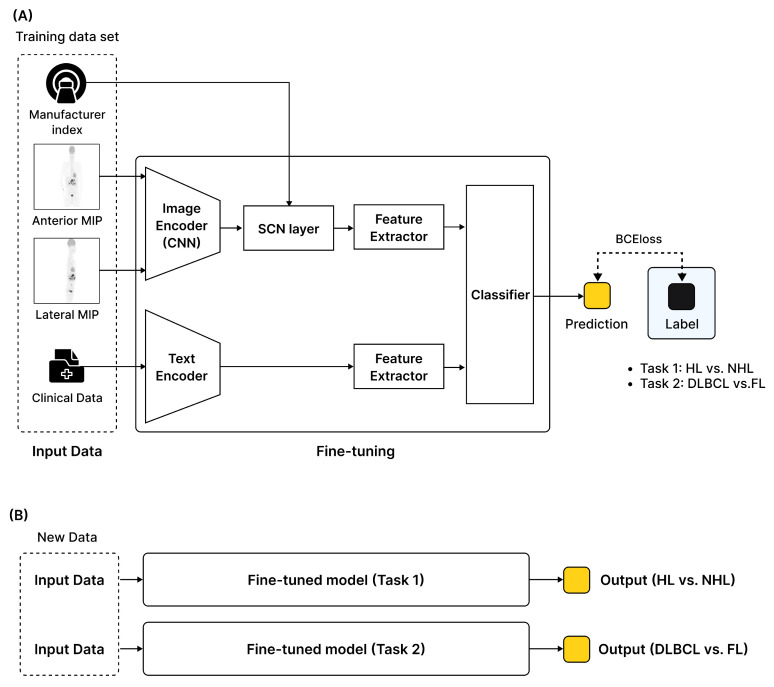
Workflow of the proposed multimodal deep learning pipeline. (**A**) Training workflow of the deep learning model. (**B**) Inference process for predicting lymphoma subtypes. Abbreviation: MIP, maximum intensity projection; CNN, convolutional neural network; SCN, scanner-conditional normalization; HL, Hodgkin lymphoma; NHL, non-Hodgkin lymphoma; DLBCL, diffuse large B-cell lymphoma; FL, follicular lymphoma.

The clinical variables are input into the clinical feature extractor, where they were transformed into clinical embedding vectors using three fully connected layers with activation functions. The image and clinical embedding vectors are then concatenated to form a single unified embedding vector. This integrated vector is passed on to the final classifier to determine the subtype. The overall architecture of LymphoMAP is shown in Figure 3. The model was trained using a binary cross-entropy loss function and optimized using the AdamW optimizer. To prevent overfitting and achieve optimal performance, early stopping and learning rate scheduling techniques were applied during training. In addition, hyperparameter optimization was conducted using a grid search. Each combination of hyperparameters was evaluated using the internal validation performance, and the configuration yielding the best results was selected as the final model setting. LymphoMAP was applied to two lymphoma classification tasks: distinguishing (1) HL from NHL and (2) DLBCL from FL. In this study, class imbalance was present in the subtype classification problem; therefore, class weights were applied to the loss function to compensate for minority classes. All model training and development were performed in an Ubuntu 18.04 environment, using the Pandas (1.5.3), Scikit-learn (1.2.1), NumPy (1.23.5), Matplotlib (3.6.3), PyTorch (1.13.1), and OpenCV-python (4.7.0.68) packages. The experiments were conducted on a GPU server equipped with four NVIDIA Tesla V100 (32 GB) GPUs and an Intel Xeon Gold 6248 CPU.

### 2.4. Statistical Analysis

Descriptive statistics were used to summarize the data. Categorical variables are presented as frequencies and percentages, and group comparisons were performed using the chi-square test. Continuous variables are presented as the mean ± standard deviation, and group comparisons were conducted using analysis of variance.

The performance of the deep learning model was evaluated based on objective metrics, including specificity, sensitivity, and the Matthews correlation coefficient (MCC). The area under the curve (AUC) of the receiver operating characteristic (ROC) curve was calculated to evaluate the binary classification performance [17]. ROC curves were plotted to provide a visual comparison of classification performance across internal and external test cohorts, and confusion matrices were constructed to summarize class-wise prediction outcomes. Statistical comparisons of AUC values were performed using two distinct methods depending on the nature of the comparison. DeLong’s test was employed to compare performance between independent cohorts (i.e., internal vs. external test sets). Conversely, to compare the performance of different models within the same cohort (e.g., ablation studies and input modality combinations), a bootstrap-based test (resampling *n* = 1000) was applied to estimate the empirical 95% confidence interval (CI) of the differences and the two-sided *p* value [18]. To provide visual interpretability of the model’s decision-making process, gradient-weighted class activation mapping (Grad-CAM) was used to highlight the discriminative regions contributing to model predictions [19].

## 3. Results

### 3.1. Clinical Characteristics of the Patients

A total of 840 patients were included in the training cohort, while 211 and 373 patients were included in the internal and external test cohorts, respectively. The demographic and clinical characteristics of each cohort are summarized in Table 1. The distribution of lymphoma subtypes differed across the cohorts, with DLBCL being the most common subtype in all cohorts. No significant differences were observed in age, sex, WBC count, ANC, or Hb among the three cohorts. However, statistically significant differences were observed in the site of involvement, Ann Arbor stage, LDH level, ALC, and PLT count. In all cohorts, the head and neck region was the most frequently involved site, and stage IV disease was the most prevalent.

### 3.2. Performances of LymphoMAP

Among the pretrained CNN models evaluated for image feature extraction in LymphoMAP, the ConvNeXt-Small model was selected as the backbone. Detailed classification results for all evaluated backbone models are provided in Supplementary Table S3. LymphoMAP was applied to two lymphoma classification tasks, and the performance metrics for each task are summarized in Table 2. We evaluated performance using combined anterior/lateral MIP images and clinical variables. In the classification between HL and NHL, the model demonstrated robust performance with an AUC of 0.89 (95% CI: 0.78–0.96) for the internal test and 0.84 (95% CI: 0.76–0.92) for the external test. Notably, the Specificity remained stable at 0.76 in both cohorts, although a slight decrease in Sensitivity was observed in the external test (0.87 vs. 0.77). For the classification between DLBCL and FL, the internal test AUC was 0.84 (95% CI: 0.74–0.92), and the external test AUC was 0.76 (95% CI: 0.67–0.84). In terms of the Matthews Correlation Coefficient (MCC), which serves as a robust metric for imbalanced datasets, the model achieved 0.43 and 0.39 in the internal tests for each task, respectively, indicating a substantial correlation between prediction and ground truth. Bootstrap-based statistical analysis comparing the AUCs of the internal and external tests for both classification tasks demonstrated no statistically significant differences (*p* > 0.05; CIs included zero). In the external DLBCL–FL task, specificity decreased to 0.65 (vs. 0.83 internal), with a wider confidence interval (0.48–0.81). This increased uncertainty likely results from the small number of FL cases in the external cohort.

**Figure 4:**
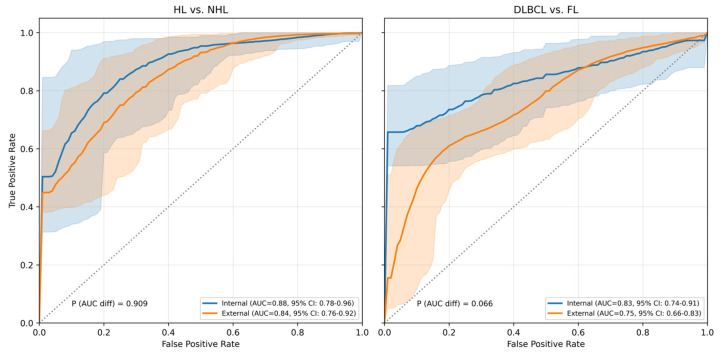
ROC curves of subtype classification in internal and external cohorts. Abbreviation: ROC, receiver operating characteristic; AUC, area under curve; HL, Hodgkin lymphoma; NHL, non-Hodgkin lymphoma; DLBCL, diffuse large B-cell lymphoma; FL, follicular lymphoma.

Figure 4 illustrates the ROC curves for the two classification tasks. In the classification between HL and NHL (Left), the ROC curves for the internal and external cohorts were nearly superimposed, with a DeLong’s test *p*-value of 0.909, confirming the model’s high stability across datasets. Similarly, regarding the classification between DLBCL and FL (Right), although a visual separation was observed between the internal and external curves, the difference in AUCs was not statistically significant (*p* = 0.066). The wider confidence intervals in the external cohort reflect uncertainty from the small sample size. However, the lack of significant performance degradation suggests that the model remains effective on unseen data. Detailed confusion matrices for both classification tasks are provided in Supplementary Figure S1, offering a comprehensive breakdown of correct and incorrect predictions across cohorts.

### 3.3. Impact of SCN on Model Performance

**Figure 5:**
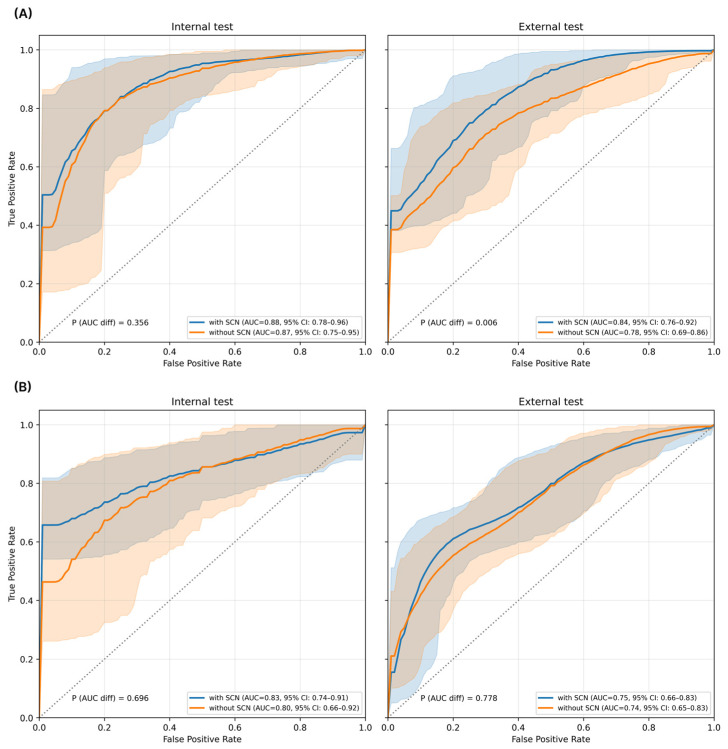
Comparison of ROC curves with and without the SCN module. (**A**) Classification between HL and NHL. (**B**) Classification between DLBCL and FL. Abbreviation: ROC, receiver operating characteristic; SCN, scanner-conditioned normalization; AUC, area under curve; HL, Hodgkin lymphoma; NHL, non-Hodgkin lymphoma; DLBCL, diffuse large B-cell lymphoma; FL, follicular lymphoma.

To evaluate the effectiveness of the SCN module in mitigating domain shifts caused by different PET/CT scanner manufacturers, we compared the model’s classification performance with and without the SCN component. As summarized in Table 3 and shown in Figure 5, the model without SCN exhibited reduced performance when evaluated on the external test cohort, indicating decreased robustness to scanner-related domain shifts. In the classification between HL and NHL, the AUC decreased from 0.87 (95% CI, 0.75–0.95) in the internal test to 0.78 (95% CI, 0.68–0.87) in the external test. This performance improvement attributable to the SCN module was statistically significant based on the bootstrap-based test (*p* < 0.05). In contrast, performance degradation was less pronounced in the internal cohort, suggesting that scanner-related variability primarily affected cross-site generalization. These results indicate that the SCN module contributes to improved robustness against site-specific scanner differences.

### 3.4. Impact of Input Modalities on Performance

**Figure 6:**
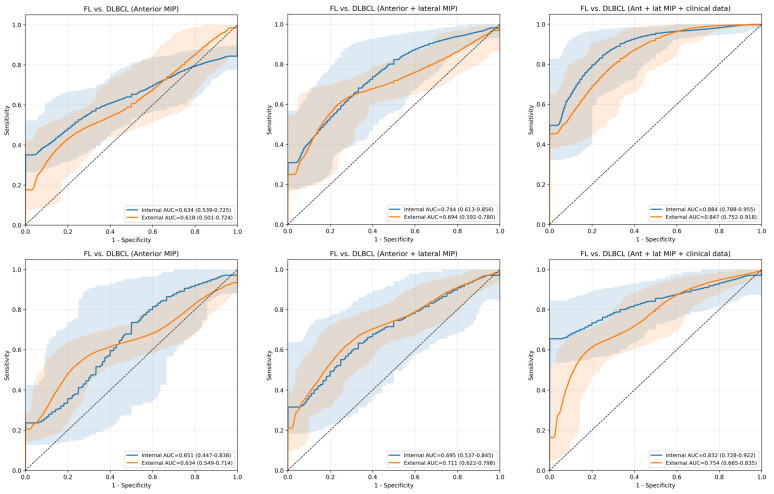
ROC curves demonstrating the incremental impact of input modalities on classification performance. The curves illustrate the stepwise performance improvement in the internal (blue) and external (orange) test cohorts as input data is enriched: Anterior MIP only (**Left**), Anterior and Lateral MIPs (**Center**), and the combined model with clinical data (**Right**). The top row represents the classification between HL and NHL, while the bottom row represents the classification between DLBCL and FL. Abbreviation: ROC, receiver operating characteristic; MIP, maximum intensity projection; AUC, area under curve; HL, Hodgkin lymphoma; NHL, non-Hodgkin lymphoma; DLBCL, diffuse large B-cell lymphoma; FL, follicular lymphoma.

The contribution of each input modality to the classification performance was systematically evaluated. All experiments were conducted under identical model architectures and training protocols to ensure fair comparison, in line with established fair benchmarking practices [16], and differences in AUC values were assessed using a bootstrap-based statistical test [20]. For HL versus NHL classification in the internal test cohort, the model trained with anterior MIP images alone yielded an AUC of 0.63 (95% CI, 0.54–0.72). Performance improved with the addition of lateral MIP images (AUC, 0.77; 95% CI, 0.66–0.88). The best performance was observed when clinical variables were incorporated, yielding an AUC of 0.89 (95% CI, 0.78–0.96), which was significantly higher than that of the anterior MIP-only model (*p* < 0.05). A similar trend was observed in the external test cohort, where the anterior MIP-only model yielded an AUC of 0.62 (95% CI, 0.51–0.73). The inclusion of lateral MIP images resulted in a marginal improvement (AUC, 0.64; 95% CI, 0.52–0.75), while the addition of clinical variables significantly increased the performance to an AUC of 0.84 (95% CI, 0.76–0.92). For DLBCL versus FL classification, the internal test demonstrated incremental performance gains with the integration of multi-view imaging and clinical data, with AUCs of 0.64 (95% CI, 0.55–0.72) for anterior MIP-only, 0.70 (95% CI, 0.54–0.86) for combined anterior and lateral MIPs, and 0.84 (95% CI, 0.74–0.92) for the multimodal model. Similarly, in the external test cohort, the AUC increased from 0.66 (95% CI, 0.45–0.83) using anterior MIPs alone to 0.71 (95% CI, 0.62–0.79) with dual-view MIPs and further to 0.76 (95% CI, 0.67–0.84) with the inclusion of clinical variables. Detailed performance metrics for each combination are provided in Supplementary Table S4. Figure 6 visually demonstrates the incremental improvement in AUC as additional modalities are integrated.

### 3.5. Grad-CAM Visualization

**Figure 7:**
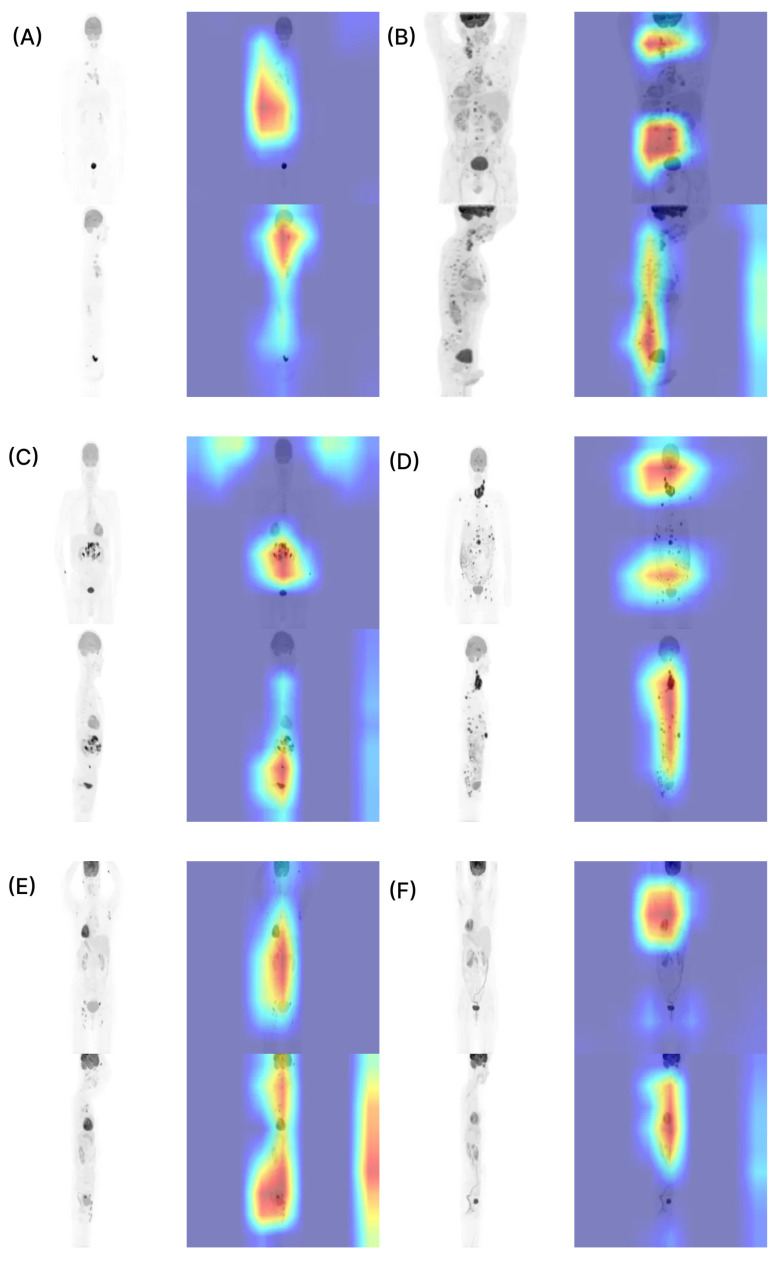
Model performance visualization using Grad-CAM. (**A**) Thoracic lymph node involvement, Ann Arbor stage I; Deauville score 5 in the supradiaphragmatic region; cHL. (**B**) Head and neck lymph node and bone marrow involvement, Ann Arbor stage IVB; Deauville scores 5 in the supradiaphragmatic and bone marrow; cHL. (**C**) Abdominal lymph node involvement, Ann Arbor stage I; Deauville score 5 in both supradiaphragmatic and infradiaphragmatic regions; DLBCL. (**D**) Head and neck lymph node involvement, Ann Arbor stage IV; Deauville score 5 in the supradiaphragmatic region; DLBCL. (**E**) Pelvic and supradiaphragmatic lymph node involvement, Ann Arbor stage IV; Deauville score 5 in both supradiaphragmatic and infradiaphragmatic regions; FL. (**F**) Abdominal lymph node involvement, Ann Arbor stage IV; Deauville score 5 in the infradiaphragmatic region; FL. Colors indicate the importance of image regions for the model’s prediction, with warmer colors (red) representing higher contribution and cooler colors (blue) lower contribution. Abbreviation: cHL, classical Hodgkin lymphoma; DLBCL, diffuse large B-cell lymphoma; Grad-CAM, gradient-weighted class activation mapping; FL, follicular lymphoma.

To interpret the decision-making process of the model for lymphoma subtype classification, we visualized the MIP images using Grad-CAM. Figure 7 presents representative cases encompassing various histological subtypes (HL, DLBCL, and FL), sites of involvement, and Ann Arbor stages. Grad-CAM visualization consistently demonstrated that the model predominantly focused on the regions corresponding to lymphoma involvement in all cases. Specifically, the highlighted areas aligned well with the anatomically involved sites identified in clinical assessments, such as the lymph nodes and bone marrow, with high metabolic activity. Furthermore, the extent and distribution of these highlighted regions varied according to disease stage. In early-stage cases, the focus was relatively localized to specific nodal sites, whereas in advanced cases, the model focused on widespread hypermetabolic areas spanning both supradiaphragmatic and infradiaphragmatic regions, reflecting the systemic nature of disease dissemination.

## 4. Discussion

In this retrospective multi-center study involving 1424 participants, we developed LymphoMAP, a deep learning-based model integrating ^18^F-FDG PET MIP images and clinical data, to automatically classify lymphoma subtypes. The model, based on the same architecture, was independently trained on two binary classification tasks (HL versus NHL and DLBCL versus FL). Both internal and external tests demonstrated high performance in the classification of HL and NHL, with an AUC above 0.85. For the classification of DLBCL and FL, the model showed a similar performance in the internal test and a relatively lower but still acceptable classification performance in the external test. In particular, external classification of DLBCL versus FL was characterized by reduced specificity and a wide confidence interval (95% CI, 0.48–0.81), indicating that the estimate was statistically unstable and susceptible to the limited FL sample size. However, since HL classification remained stable despite similar constraints, this decline suggests an influence from the intrinsic biological ambiguity between DLBCL and FL, rather than sample size alone. FL and DLBCL exist on a biological continuum. FL frequently transforms into DLBCL, and grade 3B FL often shows intermediate patterns. These factors blur the boundary between indolent and aggressive diseases. Studies also show a substantial molecular overlap between transformed FL and de novo DLBCL. Such shared origins can reduce the separability of these subtypes in imaging [21]. In contrast, HL is defined by pathognomonic Reed–Sternberg cells. Its clear biological distinctions from NHL likely contribute to more stable classification performance across cohorts. Importantly, there were no statistically significant differences in model performance between the internal and external test cohorts across any of the classification tasks. This indicates that LymphoMAP operates without significant performance degradation in the external tests, suggesting the model’s robustness and generalizability. Grad-CAM was applied to interpret the model’s classification basis, which revealed that the model primarily focused on FDG-avid lesions and clinically important involvement sites. Patients with higher Ann Arbor stages showed stronger responses to widespread lesions. This suggests the model reflects actual tumor burden and spread.

In addition to imaging data, we integrated widely used clinical variables such as age, LDH level, and Ann Arbor stage into the model. Although blood-based indicators such as WBC count, RBC count, Hb, PLT, ANC, ALC, NLR, and PLR are not direct diagnostic criteria for lymphoma subtypes, they can indirectly reflect the biological characteristics of the disease and provide supplementary information for risk stratification and prognosis. These variables may not distinguish subtypes independently, but they can help delineate prognosis within the same subtype group [22]. For instance, among patients diagnosed with DLBCL, those with a higher NLR may be at a greater risk of recurrence. In our study, the systematic evaluation of input modalities demonstrated a stepwise performance improvement: while the dual-view MIP approach enhanced the AUC compared to the single-view baseline by recovering spatial context [16], the integration of clinical variables yielded the most substantial gain, achieving a statistically significant improvement over image-only models (*p* < 0.05). This result suggests that clinical variables provided orthogonal information that complemented the imaging features, thereby enhancing the model’s discriminatory power. Recent studies have also reported improved predictive performance by integrating clinical data into image-based deep learning models [23,24]. Therefore, this study empirically supports the potential benefit of combining clinical and imaging data in lymphoma subtype classification on a multi-center scale. Future research should expand this approach by developing prognostic AI models that leverage these clinical indicators to further refine outcome predictions in larger multi-institutional cohorts.

Research on lymphoma subtype classification based on PET imaging has increased recently. Noting that metabolic patterns differ by subtype, several studies have reported methods to distinguish between aggressive and indolent NHL or to classify DLBCL and FL using PET/CT images [6,25,26]. However, most existing studies rely on radiomics-based approaches, which require precise lesion segmentation, a process that involves interobserver variability, high time and cost demands, and significant complexity. To overcome these limitations, deep learning-based approaches are being increasingly explored. For example, Diao et al. addressed the issue of limited labels by incorporating two-dimensional (2D) region of interest (ROI) segmentation and image reconstruction as auxiliary tasks and enhanced performance by integrating radiomic and deep learning features [27]. Similarly, Xu et al. used a few-shot learning approach to mitigate the challenge of small medical datasets, extracting radiomic features from PET/CT images, and combining them with deep learning features [28]. However, these studies were mostly conducted using data from single institutions, and the generalizability and reproducibility of the models have not been sufficiently validated.

PET images exhibit structural heterogeneity due to differences in scanner manufacturers, reconstruction algorithms, and scanning protocols across hospitals. This variability can cause deep learning models to overfit institutional or equipment-specific data, thereby limiting the reliability and reproducibility of AI in clinical practice. Therefore, evaluating the generalizability of models across diverse datasets is essential [29]. Recently, deep learning-based extensions like DeepComBat have been developed to effectively remove complex and nonlinear batch effects [30]. Building on this advancement, we implemented an SCN module to directly integrate harmonization within the CNN architecture. Unlike the classical post-hoc ComBat approach that adjusts features statically after extraction, SCN is designed as a trainable module that enables end-to-end optimization. This allows the model to learn scanner-invariant representations by jointly updating the feature extractor and harmonization parameters. Indeed, our ablation study demonstrated that the inclusion of SCN contributed to improved performance on the external dataset, suggesting that this dynamic harmonization strategy effectively mitigates scanner-dependent domain shifts.

Furthermore, this study designed a model to automatically learn the key metabolic patterns necessary for lymphoma subtype classification without requiring separate lesion ROI annotations. The three-dimensional (3D) PET images were converted into MIP format to visually summarize the metabolic distribution of lesions, reduce the preprocessing burden, and improve computational efficiency. Notably, using only two MIP images (anterior and lateral) per patient, we minimized the data volume while maintaining high performance. Previous studies have typically used both PET and CT images or employed multiple 2D slices in the axial, coronal, and sagittal directions or even entire 3D volumes as input [27,28]. In contrast, despite the simplified input structure, our model outperformed previous studies, such as those by Dian et al. for DLBCL versus HL classification (AUC 0.75) and Xu et al. for DLBCL versus FL classification (AUC 0.79). These results demonstrate that our simplified MIP-based input is sufficient to achieve a high performance in subtype classification, effectively learning metabolic information without complex lesion segmentation [31]. This approach enhances the efficiency of data construction and processing while also highlighting the practical advantages of a deep learning-based lymphoma classification strategy with greater applicability and scalability in real-world clinical settings.

Lymphoma subtypes differ significantly in treatment strategy, drug selection, treatment intensity, and prognosis [32]. While early intensive treatment is essential for certain high-risk subtypes, avoiding unnecessary treatment is crucial for low-risk subtypes. The proposed LymphoMAP model demonstrates the potential to non-invasively classify lymphoma subtypes using a relatively small number of routinely acquired ^18^F-FDG PET images. By enabling rapid subtype prediction without invasive procedures or manual processes such as tumor contouring or ROI selection, the model provides fully automated inference immediately after image acquisition. This non-invasive prediction capability could be particularly beneficial as a complementary aid in clinical scenarios where pathological diagnosis is delayed, tissue biopsy is technically challenging or inaccessible, or early subtype-specific treatment decisions are critical. By providing preliminary subtype information prior to definitive pathology, the model aims to assist clinicians in early risk assessment, prioritizing diagnostic procedures, and formulating initial treatment plans. However, it is important to note that this model is not intended to replace pathological diagnosis. This study represents an initial step, and large-scale multi-center cohorts and prospective clinical studies are necessary to further verify the robustness, generalizability, and clinical utility of the proposed approach before its routine implementation.

This study has some limitations. First, data composition limitations exist in terms of class imbalance and subtype coverage. The number of samples across lymphoma subtypes was imbalanced, and some rare subtypes were underrepresented, which may have reduced classification performance, particularly sensitivity or recall for minority classes. Although this imbalance reflects real-world prevalence, we addressed it by applying class-weighted loss to enhance the influence of minority classes during training. In addition, the current cohort did not include certain aggressive subtypes, such as Mantle Cell Lymphoma and T-cell lymphomas, limiting the scope of generalization. Future studies will expand the dataset to include a broader spectrum of lymphoma subtypes. Second, this study used a limited number of clinical variables, such as age, LDH level, and disease stage, and did not incorporate a broader range of clinical indicators associated with lymphoma. Future studies should aim to integrate more comprehensive clinical and biological data to further enhance model performance and interpretability.

Third, the use of 2D MIP images introduces inherent representational limitations. Compared with full 3D PET volumes, MIP projections inevitably lose depth information, which can lead to underestimation of lesion extent and inaccurate depiction of lesion-to-lesion relationships [33]. In particular, tumors located along the same line of projection may appear merged into a single focus, while small or low-uptake lesions can be masked by adjacent intensely hypermetabolic regions. Although a multi-view strategy using anterior and lateral projections was employed to partially mitigate this limitation, this approach still relies on a limited subset of views and cannot fully recover the lost 3D context. Future studies should therefore investigate hybrid pipelines that retain the computational efficiency of MIP inputs while selectively incorporating volumetric information. Fourth, regarding the harmonization strategy, although the SCN module effectively mitigates domain shifts arising from different scanner manufacturers, it relies on learned manufacturer-specific embeddings. Consequently, the current model may not optimally generalize to data acquired from scanner manufacturers that were completely absent during the training phase, which would require retraining or fine-tuning. Fifth, despite the inclusion of multi-institutional external validation and scanner-aware harmonization, all data were obtained from a single country. Consequently, the model’s generalizability across different healthcare systems and international imaging practices remains to be validated. Future studies should therefore include datasets from other countries to confirm the robustness and global applicability of the proposed framework.

## 5. Conclusions

In this study, we developed LymphoMAP, a deep learning-based model for the automated classification of lymphoma subtypes, using large-scale multi-center ^18^F-FDG PET data. By integrating MIP images from non-invasively acquired ^18^F-FDG PET scans with clinical data, the model demonstrated strong performance in distinguishing between HL and NHL, as well as between DLBCL and FL subtypes. For DLBCL versus FL classification, the model maintained comparable internal performance and demonstrated acceptable generalizability in the external cohort. Importantly, no statistically significant differences in AUC were observed between the internal and external tests for any classification task, suggesting that LymphoMAP maintains stable performance across diverse clinical settings.

Notably, the integration of an SCN module into the deep learning pipeline effectively mitigated inter-institutional variability, contributing to the model’s robustness against scanner-dependent domain shifts. Furthermore, the model was designed to automatically learn key metabolic patterns directly from PET MIP images. This approach eliminates the need for explicit lesion segmentation or manual region-of-interest definition, thereby reducing preprocessing complexity and improving practical feasibility.

Since lymphoma subtypes differ substantially in treatment strategy, therapeutic intensity, and prognosis, providing rapid and non-invasive subtype predictions from routinely acquired imaging offers significant complementary value in clinical workflows. In particular, LymphoMAP may serve as a supportive tool in scenarios where pathological confirmation is delayed or tissue acquisition is technically challenging, assisting in early risk stratification and the prioritization of diagnostic procedures. However, this model is not intended to replace histopathological diagnosis, and its outputs should be interpreted as adjunctive information. Further validation in larger multi-center cohorts and prospective clinical studies is required to fully establish the clinical utility and reliability of the proposed approach.

## Figures and Tables

**Table 1:** Patient population characteristics.

Patient Characteristics		Training Cohort (*n* = 840)	Internal Test Cohort (*n* = 211)	External Test Cohort (*n* = 373)	*p* Value
Age (Years), mean ± SD		67 ± 16	68 ± 15	68 ± 15	0.55
Sex, *n* (%)					0.17
	Male	491 (58%)	114 (54%)	231 (62%)	
	female	349 (42%)	97 (46%)	142 (38%)	
Site of involvement, *n* (%)					<0.05
	Head and neck	245 (29%)	58 (27%)	142 (38%)	
	Thoracic	51 (6%)	13 (6%)	32 (9%)	
	Abdominal	155 (18%)	40 (19%)	63 (17%)	
	Pelvis	54 (6%)	15 (7%)	22 (6%)	
	Etc.	200 (24%)	52 (25%)	114 (31%)	
	N/A	135 (16%)	33 (16%)	0 (0%)	
Ann Arbor stage, *n* (%)					<0.05
	I	118 (14%)	33 (16%)	56 (15%)	
	II	172 (20%)	52 (25%)	108 (29%)	
	III	148 (18%)	39 (18%)	55 (15%)	
	IV	402 (48%)	87 (41%)	154 (41%)	
Histologic subtype, *n* (%)					<0.05
	HL	65 (8%)	16 (8%)	21 (6%)	
	DLBCL	334 (40%)	87 (41%)	173 (46%)	
	FL	48 (6%)	7 (3%)	34 (9%)	
	Etc.	328 (46%)	85 (48%)	145 (39%)	
LDH (IU/L), mean ± SD		381 ± 445	365 ± 350	314 ± 240	<0.05

Abbreviation: HL, Hodgkin lymphoma; DLBCL, diffuse large B cell lymphoma; FL, follicular lymphoma; LDH, lactate dehydrogenase.

**Table 2:** Performance of LymphoMAP for lymphoma subtype classification.

Classification	Cohort	AUC (95% CI)	MCC (95% CI)	Sensitivity (95% CI)	Specificity (95% CI)
HL vs. NHL	Internal test cohort	0.89 (0.78, 0.96)	0.43 (0.26, 0.58)	0.87 (0.82, 0.91)	0.76 (0.54, 0.94)
	External test cohort	0.84 (0.76, 0.92)	0.28 (0.16, 0.38)	0.77 (0.72, 0.81)	0.76 (0.56, 0.93)
DLBCL vs. FL	Internal test cohort	0.84 (0.74, 0.92)	0.39 (0.17, 0.56)	0.74 (0.65, 0.83)	0.83 (0.56, 1.0)
	External test cohort	0.76 (0.67, 0.84)	0.25 (0.11, 0.38)	0.68 (0.48, 0.75)	0.65 (0.48, 0.81)

Abbreviation: AUC, area under curve; MCC, Matthews correlation coefficient; CI, confidence interval; HL, Hodgkin lymphoma; NHL, non-Hodgkin lymphoma; DLBCL, diffuse large B-cell lymphoma; FL, follicular lymphoma.

**Table 3:** Ablation study demonstrating the impact of the SCN module on classification performance.

Classification	Cohort	AUC (95% CI)	MCC (95% CI)	Sensitivity (95% CI)	Specificity (95% CI)
HL vs. NHL	Internal test cohort	0.87 (0.75, 0.95)	0.41 (0.23, 0.56)	0.83 (0.80, 0.88)	0.82 (0.60, 1.0)
	External test cohort	0.78 (0.68, 0.87)	0.21 (0.07, 0.33)	0.81 (0.76, 0.84)	0.58 (0.35, 0.79)
DLBCL vs. FL	Internal test cohort	0.81 (0.67, 0.92)	0.26 (0.04, 0.47)	0.74 (0.65, 0.83)	0.64 (0.38, 0.9)
	External test cohort	0.74 (0.66, 0.83)	0.25 (0.10, 0.39)	0.72 (0.66,0.77)	0.59 (0.42, 0.75)

The baseline model represents architecture trained without the SCN module. Abbreviation: AUC, area under curve; MCC, Matthews correlation coefficient; CI, confidence interval; HL, Hodgkin lymphoma; NHL, non-Hodgkin lymphoma; DLBCL, diffuse large B-cell lymphoma; FL, follicular lymphoma.

## Data Availability

The datasets generated and/or analyzed in this study are available from the corresponding author upon reasonable request.

## Supplementary Materials

The following supporting information can be downloaded at https://www.mdpi.com/article/10.3390/cancers18020210/s1, Table S1. Data completeness. Table S2. Summary of PET/CT scanners. Table S3. Summary of PET/CT scanners. Table S4. Performance of CNN models. Figure S1. Confusion matrix of subtype classification in internal and external cohorts.

## Abbreviations

The following abbreviations are used in this manuscript:

^18^F-FDG	Fluorine-18 fluorodeoxyglucose
PET	Positron emission tomography
MIP	Maximum intensity projection
HL	Hodgkin lymphoma
NHL	Non-Hodgkin lymphoma
DLBCL	Diffuse large B-cell lymphoma
FL	Follicular lymphoma
PNUH	Pusan National University Hospital
DICOM	Digital Imaging and Communication in Medicine
CT	Computed tomography
WBC	White blood cell count
ANC	Absolute neutrophil count
ALC	Absolute lymphocyte count
PLT	Platelet count
Hb	Hemoglobin level
NLR	Neutrophil-to-lymphocyte ratio
PLR	Platelet-to-lymphocyte ratio
LDH	Lactate dehydrogenase
CNN	Convolutional neural network
MCC	Matthews correlation coefficient
AUC	Area under the curve
ROC	Receiver operating characteristic
CI	Confidence interval
Grad-CAM	Gradient-weighted class activation mapping
2D	Two-dimensional
3D	Three-dimensional
ROI	Region of interest
