# Supplementary material for: Robust Multimodal Deep Learning for Lymphoma Subtype Classification Using 18F-FDG PET Maximum Intensity Projection Images and Clinical Data: A Multi-Center Study
Source: Cancers (Basel). 2026 Jan 9;18(2):210. doi: 10.3390/cancers18020210 (PMC12838601; doi:10.3390/cancers18020210)
Supplement: Supplementary file 1 [file cancers-18-00210-s001.zip › cancers-4043985-supplementary.pdf]

## Supplementary Tables

**Supplementary Table S1.** Data completeness

| | AUMC (n = 402) | CNUH (n = 163) | EUMC (n = 168) | HUMC (n = 183) | PNUH (n = 373) | SCHMC (n = 135) |
|---|---|---|---|---|---|---|
| Age | 100.0% | 100.0% | 100.0% | 100.0% | 100.0% | 100.0% |
| Sex | 100.0% | 100.0% | 100.0% | 100.0% | 100.0% | 100.0% |
| Diabetes | 99.8% | 100.0% | 100.0% | 97.3% | 100.0% | 100.0% |
| Hypertension | 99.8% | 100.0% | 100.0% | 96.7% | 100.0% | 100.0% |
| Smoking | 96.5% | 100.0% | 0.0% | 89.1% | 100.0% | 100.0% |
| Family history | 98.5% | 100.0% | 0.0% | 94.5 | 100.0% | 100.0% |
| Location cancer | 100.0% | 100.0% | 0.0% | 100.0% | 100.0% | 100.0% |
| Height | 99.5% | 100.0% | 99.4% | 100.0% | 100.0% | 100.0% |
| weight | 100.0% | 100.0% | 100.0% | 100.0% | 100.0% | 100.0% |
| WBC | 100.0% | 100.0% | 100.0% | 100.0% | 100.0% | 100.0% |
| ANC | 100.0% | 100.0% | 100.0% | 100.0% | 100.0% | 100.0% |
| ALC | 100.0% | 100.0% | 100.0% | 100.0% | 100.0% | 100.0% |
| PLT | 100.0% | 100.0% | 100.0% | 100.0% | 100.0% | 100.0% |
| Hb | 100.0% | 100.0% | 100.0% | 100.0% | 100.0% | 100.0% |
| NLR | 100.0% | 100.0% | 100.0% | 100.0% | 100.0% | 100.0% |
| PLR | 100.0% | 100.0% | 100.0% | 100.0% | 100.0% | 100.0% |
| LDH | 100.0% | 100.0% | 100.0% | 100.0% | 100.0% | 100.0% |

Overview of data completeness across institutions. Among the two institutions with complete data (100% completeness), the one with a higher sample size was selected for external validation.

AUMC, Ajou university medical center; CNUH, Chungnam national university hospital; EUMC, Ewha womans university medical center; HUMC, Hallym university medical center; PNUH, Pusan national university hospital; SCHMC, Soonchunhyang university hospital.

**Supplementary Table S2.** Summary of PET/CT scanners.

| Institution | Manufacturer | Model Name | $^{18}$F-FDG dose (MBq), median (IQR) | Slice thickness (mm) | Number of patients |
|---|---|---|---|---|---|
| AUMC | GE MEDICAL SYSTEMS | Discovery ST | 391.46 (170.98-771.32) | 3.27 | 212 |
| AUMC | GE MEDICAL SYSTEMS | Discovery STE | 375.79 (-126.0-770.09) | 3.27 | 188 |
| AUMC | Philips Medical Systems | Allegro Body(C) | 284.9 (284.9-284.9) | 4.0 | 1 |
| AUMC | Philips Medical Systems | GEMINI TF TOF 16 | 299.7 (299.7-299.7) | 4.0 | 1 |
| CNUH | GE MEDICAL SYSTEMS | Discovery 690 | 223.23 (132.31-327.18) | 3.27 | 154 |
| CNUH | GE MEDICAL SYSTEMS | Discovery ST | 226.47 (216.55-242.62) | 3.27 | 3 |
| CNUH | SIEMENS | Biograph TruePoint TrueV PET/CT | 362.6 (362.6-362.6) | 5 | 1 |
| CNUH | SIEMENS | Biograph 128 mCT | 256.04 (192.4-307.1) | 5 | 5 |
| EUMC | GE MEDICAL SYSTEMS | Discovery MI | 182.77 (151.58-213.1) | 2.79 | 10 |
| EUMC | SIEMENS | Biograph128 | 323.58 (229.4-421.06) | 5 | 29 |
| EUMC | SIEMENS | Biograph128 mCT | 244.13 (120.25-349.65) | 3 | 129 |
| HUMC | GE MEDICAL SYSTEMS | Discovery ST | 380.4 (318.65-418.79) | 3.27 | 23 |
| HUMC | Philips Medical Systems | GEMINI TF TOF 16 | 335.17 (268.99-370.0 | 4 | 50 |
| HUMC | Philips Medical Systems | GEMINI TF TOF 64T | 308.92 (260.85-384.8 | 4 | 73 |
| HUMC | Philips Medical Systems | Gemini TF(C) | 447.88 (440.67-455.1 | 4 | 2 |
| HUMC | SIEMENS | Biograph TruePoint TrueV PET/CT | 370.0 (370.0-370.0 | 5 | 1 |
| HUMC | SIEMENS | Biograph64 mCT | 314.57 (268.99-391.83 | 5 | 32 |
| HUMC | SIEMENS | SOMATOM Definition AS mCT | 302.1 (268.62-335.59 | 5 | 2 |
| PNUH | Philips Medical Systems | GEMINI TF TOF 64 | 319.61 (240.13-418.1 | 4 | 45 |
| PNUH | SIEMENS | Biograph TruePoint TrueV PET/CT | 270.8 (218.3-321.9 | 2 | 21 |
| PNUH | SIEMENS | Biograph40 TruePoint | 329.15 (196.1-518.0 | 2 | 280 |
| SCHMC | CTI PET Systems | ECAT EXACT HR | 455.63 (388.5-710.4 | 3.375 | 14 |

| | | | | | |
|---|---|---|---|---|---|
| SCHMC | SIEMENS | Biograph128 mCT | 280.04 (148.0-407.0 | 3 | 107 |
| SCHMC | SIEMENS | SOMATOM Definition AS mCT | 266.66 (185.0-358.9 | 5 | 14 |

Summary of PET/CT scanners used at participating institutions, incuding manufacturer and model, median [$^{18}$F]FDG dose (MBq), slice thickness (mm), and number of patients scanned at each site.

AUMC, Ajou university medical center; CNUH, Chungnam national university hospital; EUMC, Ewha womans university medical center; HUMC, Hallym university medical center; PNUH, Pusan national university hospital; SCHMC, Soonchunhyang university hospital.

**Supplementary Table S3**. Performance of CNN models

| Classification | Model | Internal test set | | | | External validation set | | | |
|---|---|---|---|---|---|---|---|---|---|
| | | AUC (95% CI) | F1 score (95% CI) | Sensitivity (95% CI) | Specificity (95% CI) | AUC (95% CI) | F1 score (95% CI) | Sensitivity (95% CI) | Specificity (95% CI) |
| HL vs. NHL | ResNet-50 | 0.87 (0.73,0.96) | 0.84 (0.79, 0.88) | 0.73 (0.67, 0.79) | 0.82 (0.62, 1.00) | 0.73 (0.61, 0.84) | 0.86 (0.83, 0.89) | 0.78 (0.73, 0.82) | 0.58 (0.37, 0.78) |
| HL vs. NHL | EfficientNetV2-S | 0.81 (0.67, 0.93) | 0.83 (0.78, 0.87) | 0.72 (0.65, 0.78) | 0.88 (0.70, 1.00) | 0.80 (0.69, 0.90) | 0.79 (0.75, 0.82) | 0.66 (0.61, 0.71) | 0.72 (0.50, 0.90) |
| HL vs. NHL | ConvNeXt-Small | 0.89 (0.78, 0.96) | 0.43 (0.26, 0.58) | 0.87 (0.82, 0.91) | 0.76 (0.54,0.94) | 0.84 (0.76, 0.92) | 0.28 (0.16, 0.38) | 0.77 (0.72, 0.81) | 0.76 (0.56, 0.93) |
| DLBCL vs. FL | ResNet-50 | 0.78 (0.67, 0.87) | 0.86 (0.79, 0.91) | 0.79 (0.70, 0.87) | 0.55 (0.25, 0.85) | 0.73 (0.62, 0.82) | 0.82 (0.77, 0.86) | 0.76 (0.69, 0.82) | 0.50 (0.32, 0.68) |
| DLBCL vs. FL | EfficientNetV2-S | 0.77 (0.60, 0.92) | 0.74 (0.65, 0.82) | 0.61 (0.50, 0.72) | 0.73 (0.44, 1.00) | 0.77 (0.68, 0.85) | 0.75 (0.68, 0.80) | 0.62 (0.55, 0.70) | 0.77 (0.61, 0.91) |
| DLBCL vs. FL | ConvNeXt-Small | 0.84 (0.74,0.92) | 0.39 (0.17, 0.56) | 0.74 (0.65, 0.83) | 0.83 (0.56, 1.0) | 0.76 (0.67,0.84) | 0.25 (0.11, 0.38) | 0.68 (0.48,0.75) | 0.65 (48,0.81) |

HL, hodgkin lymphoma; NHL, non-hodgkin lymphoma; DLBCL, diffuse large b cell lymphoma; FL, follicular lymphoma; AUC, Area under curve.

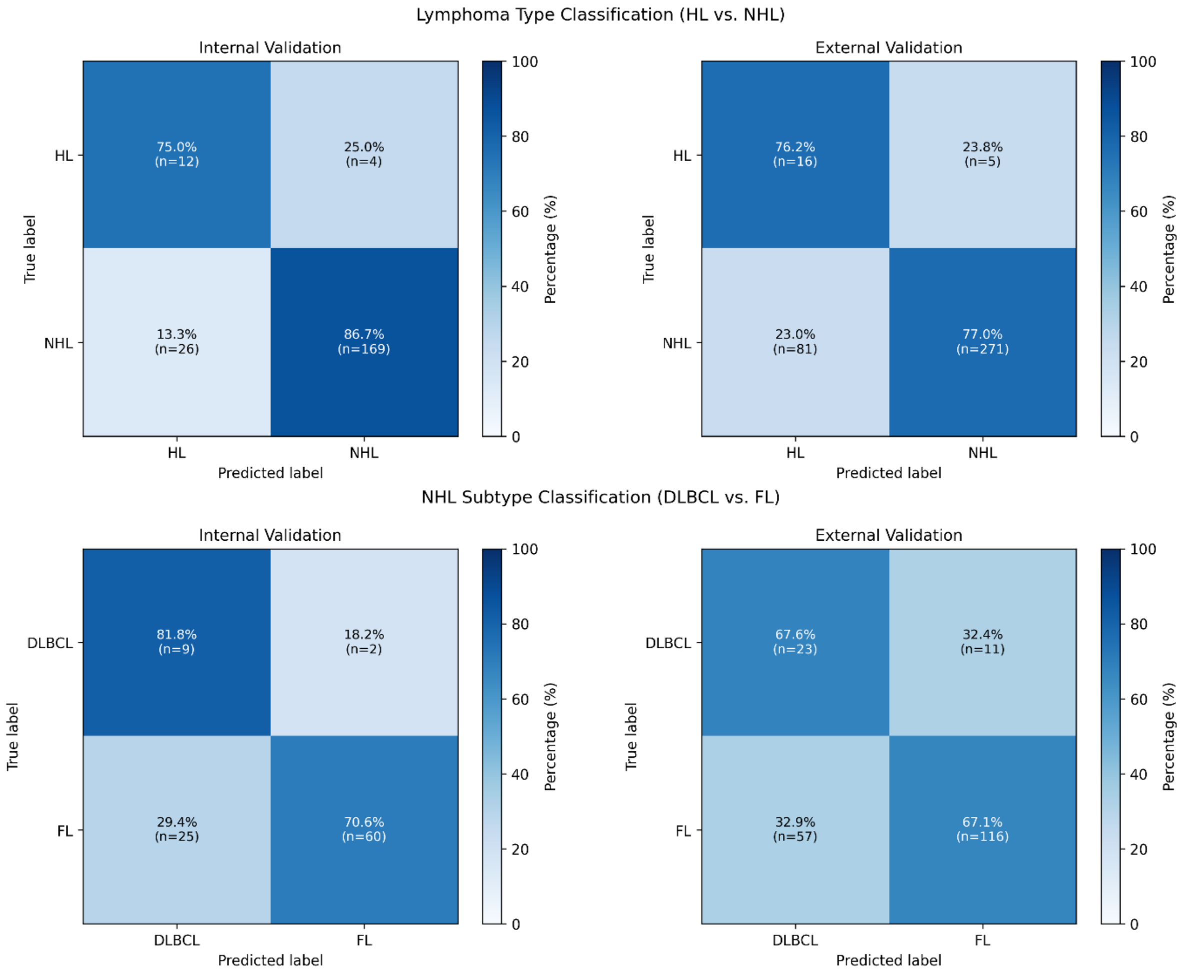


**Supplementary Figure S1.** Confusion matrix of subtype classification in internal and external cohorts. Abbreviation: HL, Hodgkin lymphoma; NHL, non-Hodgkin lymphoma; DLBCL, diffuse large B-cell lymphoma; FL, follicular lymphoma.

**Supplementary Table S4**. Performance of CNN models

| Classification | Input Data | Cohort | AUC (95% CI) | MCC (95% CI) | Sensitivity (95% CI) | Specificity (95% CI) |
|---|---|---|---|---|---|---|
| HL vs. NHL | Anterior MIP | Internal test cohort | 0.63 (0.54, 0.72) | 0.15 (0.04, 0.25) | 0.47 (0.4, 0.54) | 0.82 (0.62, 1.0) |
| HL vs. NHL | Anterior MIP | External test cohort | 0.62 (0.51, 0.73) | 0.12 (0.03, 0.19) | 0.39 (0.34, 0.44) | 0.86 (0.69, 1.0) |
| HL vs. NHL | Anterior MIP + lateral MIP | Internal test cohort | 0.77 (0.66, 0.88) | 0.21 (0.06, 0.34) | 0.73 (0.66, 0.79) | 0.63 (0.39, 0.84) |
| HL vs. NHL | Anterior MIP + lateral MIP | External test cohort | 0.64 (0.52, 0.75) | 0.08 (-0.02, 0.17) | 0.51 (0.45, 0.56) | 0.67 (0.46, 0.86) |
| HL vs. NHL | Anterior MIP + lateral MIP + clinical data | Internal test cohort | 0.89 (0.78, 0.96) | 0.43 (0.26, 0.58) | 0.87 (0.82, 0.91) | 0.76 (0.54,0.94) |
| HL vs. NHL | Anterior MIP + lateral MIP + clinical data | External test cohort | 0.84 (0.76, 0.92) | 0.28 (0.16, 0.38) | 0.77 (0.72, 0.81) | 0.76 (0.56, 0.93) |
| DLBCL vs. FL | Anterior MIP | Internal test cohort | 0.64 (0.55, 0.72) | 0.21 (0.08, 0.33) | 0.54 (0.47, 0.61) | 0.74 (0.59, 0.89) |
| DLBCL vs. FL | Anterior MIP | External test cohort | 0.66 (0.45, 0.83) | 0.1 (-0.11, 0.28) | 0.52 (0.41, 0.62) | 0.64 (0.31, 0.9) |
| DLBCL vs. FL | Anterior MIP + lateral MIP | Internal test cohort | 0.7 (0.54, 0.86) | 0.2 (-0.01, 0.38) | 0.58 (0.48, 0.68) | 0.75 (0.42, 1.0) |
| DLBCL vs. FL | Anterior MIP + lateral MIP | External test cohort | 0.71 (0.62, 0.79) | 0.23 (0.11, 0.35) | 0.58 (0.5, 0.65) | 0.74 (0.58, 0.87) |
| DLBCL vs. FL | Anterior MIP + lateral MIP + clinical data | Internal test cohort | 0.84 (0.74,0.92) | 0.39 (0.17, 0.56) | 0.74 (0.65, 0.83) | 0.83 (0.56, 1.0) |
| DLBCL vs. FL | Anterior MIP + lateral MIP + clinical data | External test cohort | 0.76 (0.67,0.84) | 0.25 (0.11, 0.38) | 0.68 (0.48,0.75) | 0.65 (48,0.81) |

Abbreviation: AUC, area under curve; MCC, Matthews correlation coefficient; HL, Hodgkin lymphoma; NHL, non-Hodgkin lymphoma; DLBCL, diffuse large B-cell lymphoma; FL, follicular lymphoma.
